# Spatiotemporal Feedback and Network Structure Drive and Encode *Caenorhabditis elegans* Locomotion

**DOI:** 10.1371/journal.pcbi.1005303

**Published:** 2017-01-11

**Authors:** James M. Kunert, Joshua L. Proctor, Steven L. Brunton, J. Nathan Kutz

**Affiliations:** 1 Department of Physics, University of Washington, Seattle, Washington, United States of America; 2 Institute for Disease Modeling, Bellevue, Washington, United States of America; 3 Department of Mechanical Engineering, University of Washington, Seattle, Washington, United States of America; 4 Department of Applied Mathematics, University of Washington, Seattle, Washington, United States of America; Oxford University, UNITED KINGDOM

## Abstract

Using a computational model of the *Caenorhabditis elegans* connectome dynamics, we show that proprioceptive feedback is necessary for sustained dynamic responses to external input. This is consistent with the lack of biophysical evidence for a central pattern generator, and recent experimental evidence that proprioception drives locomotion. The low-dimensional functional response of the *Caenorhabditis elegans* network of neurons to proprioception-like feedback is optimized by input of specific spatial wavelengths which correspond to the spatial scale of real body shape dynamics. Furthermore, we find that the motor subcircuit of the network is responsible for regulating this response, in agreement with experimental expectations. To explore how the connectomic dynamics produces the observed two-mode, oscillatory limit cycle behavior from a static fixed point, we probe the fixed point’s low-dimensional structure using Dynamic Mode Decomposition. This reveals that the nonlinear network dynamics encode six clusters of dynamic modes, with timescales spanning three orders of magnitude. Two of these six dynamic mode clusters correspond to previously-discovered behavioral modes related to locomotion. These dynamic modes and their timescales are encoded by the network’s degree distribution and specific connectivity. This suggests that behavioral dynamics are partially encoded within the connectome itself, the connectivity of which facilitates proprioceptive control.

## Author Summary

The nematode *C. elegans* lives a complex and rich life despite having only 302 neurons. The full connectivity between these neurons (its “connectome”) has been measured, making it an ideal model system for understanding how neural processing generates behavior. However, unlike most animals, it doesn’t appear to have neural circuits dedicated to generating rhythmic motion. Even the simple rhythmic behavior of forward locomotion is believed to be ultimately driven by stretch-receptive proprioception as the worm changes body shape. Computational models of connectome dynamics can capture behavioral responses, making them valuable for studying neural dynamics. We consider a model of the full-connectome dynamics which, without external input, is in a static neural state. We show that proprioception-like input can bring the system into a rhythmic state similar to locomotion. We then show that arbitrary external inputs across all neurons can only excite some combination of six neural patterns, and that the shape and dynamics of these patterns are determined by the connectome. Furthermore, two of these patterns correspond to forward locomotion, such that the connectome facilitates locomotion-like responses. We thus find that the connectome itself is not random, but is ideally tuned for generating behavior.

## Introduction

The exact process through which the nematode *Caenorhabditis elegans* (*C. elegans*) generates the rhythmic activity necessary for locomotion remains unclear [1]. In many other species, a Central Pattern Generator (CPG) is typically the source of rhythmic activity [2–6]. There is insufficient experimental evidence to support the existence of a CPG in the *C. elegans* neuronal network [7, 8]. Experimental and computational evidence shows that proprioception within motorneurons plays an important role in driving and modulating forward locomotion [9, 10], and it has been hypothesized that this proprioceptive feedback is what ultimately generates rhythmic locomotion [10], rather than any dedicated circuitry in the neuronal network. Using a computational model for the connectome dynamics of *C. elegans* [11], we provide strong theoretical and computational support, through the emerging method of dynamic mode decomposition, for the hypothesis that proprioception within motorneurons does indeed encode and drive rhythmic activity.

Critical to assessing how sustained, low-dimensional dynamic activity is generated, is understanding the role the network’s connectivity graph (its “connectome”) plays in generating rhythmic motion. The structure of a neuronal network’s connectivity often determines how the network operates as a whole [12, 13], encoding key behavioral responses characterized by low-dimensional patterns of activity [14–19]. However, the exact importance of the *specific* connectivity of a network is unclear, and neuronal network dynamics are often computationally modeled using uniform random networks [20–27]. In *C. elegans*, however, the structure of the connectome is clearly not random, and it may further play a critical role in helping to generate or facilitate rhythmic responses. This is suggested by the fact that computational models of the connectome can generate motorneuron oscillations related to forward locomotion in response to constant stimuli even without proprioception (and even when modeling neural dynamics alone, with no coupled muscular, bodily or environmental modeling) [11]. This suggests that oscillatory, stereotyped responses are, at some level, encoded within the connectome.

**Fig 1:**
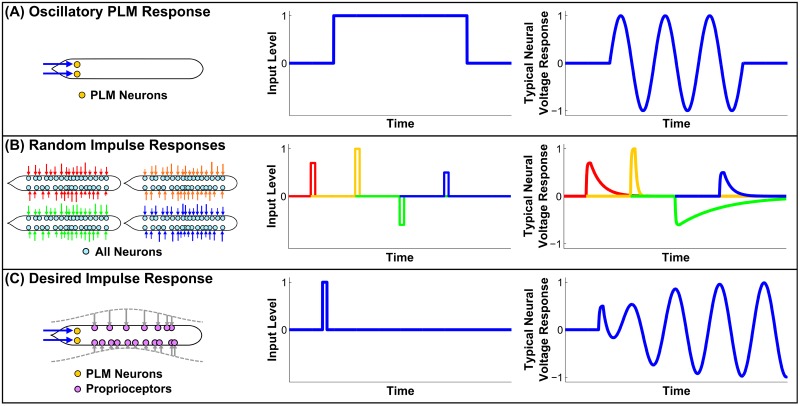
**(A)** Illustration of the oscillatory response as demonstrated in [11]. Unrealistically, the system requires constant stimulation or it will collapse into a fixed point. This is consistant with evidence that proprioceptive feedback is necessary for sustained dynamic responses to external input. **(B)** Illustration of the response to momentary random stimuli. After any stimulus, the system will decay back to the fixed point, albeit at different timescales. We will use this to probe the dynamical structure of the fixed point. **(C)** Illustration of a potentially more realistic response. We seek a mechanism for proprioceptive feedback which produces sustained responses to momentary stimuli. We will investigate the consistency of our model with such a framework.

There is, however, an important caveat to this result: oscillatory output occurs only due to an unrealistic stimulus, consisting of a constant input into the tail-touch mechanoreceptor sensory neuron pair PLM (i.e. the touch-receptive posterior lateral microtubule cells) [28]. In the absence of constant stimulus, the neural state will collapse onto a static, stable fixed point, i.e. a state of no movement. This is illustrated in Panel (A) of Fig 1. This is clearly not realistic; the actual worm is not constantly receiving tail-touch stimulus during every moment at which it crawls forward. As illustrated in Panel (B), the system will quickly decay back to static equilibrium after *any* random stimulus. A more realistic response to an impulse may perhaps look more like Panel (C): if the worm is in a pause state, a momentary stimulus should be capable of driving it into sustained motion. This lack of sustained oscillation can be explained by the model’s lack of feedback, specifically by the lack of stretch-receptive proprioception within B-class motorneurons, which is known to drive and regulate locomotion [9].

This study thus considers the following questions about the model in [11]: Is it consistent with a framework of proprioception-driven locomotion? If so, do the low-dimensional output patterns encoded by the connectome facilitate proprioceptive control? In other words, does the system’s equilibrium have a low-dimensional dynamical structure which facilitates responses related to locomotion?

In this manuscript, we demonstrate that proprioceptive feedback is indeed necessary and sufficient for sustained dynamic responses to external input. This is consistent with the lack of biophysical evidence for a central pattern generator driving locomotion, and the evidence that proprioception drives locomotion. Explicitly, we use the spatial location of specific motorneurons to drive them with a sinusoidal traveling wave, approximating strech-receptive proprioception during locomotion. The functional response of the network to this proprioception-like input is optimized by specific spatial wavelengths, specifically optimal locomotion responses are driven by input with spatial scales consistent with *C. elegans* body shape dynamics, i.e. eigenworm-like structures [29]. We then repeat this investigation for perturbed networks, including a modification in which all but the experimentally-characterized locomotion subcircuit is ablated. This reveals that the motor subcircuit alone generates a functional response nearly identical to that of the full connectome. However, we find that the locomotory motorneurons are *not* by themselves sufficient, and that locomotory interneurons are crucial to regulating the response, even though they are not stimulated directly.

By applying Dynamic Mode Decomposition to the network data, we discover that the dynamics encode six clusters of dynamic modes with timescales spanning three orders of magnitude. Two of these six dynamic mode clusters correspond to previously-discovered behavioral modes related to locomotion. The dynamic modes and timescales are encoded by the network’s degree distribution and specific connectivity. This suggests that behavioral dynamics are partially encoded within the connectome itself, the connectivity of which facilitates proprioceptive control. Thus our results suggest a framework in which the neural network is not the *source* of spontaneous oscillation, but rather is structured to facilitate specific proprioception-driven oscillation responses. More broadly, our application of Dynamic Mode Decomposition to network dynamics demonstrates its utility at discovering, from activity data alone, the responses which a network may be encoded to promote or inhibit.

## Results

### Perturbation Response and Dynamic Modes

Given the lack of evidence for a CPG within the network, it is interesting that the system is able to generate oscillation in response to a non-oscillatory input, and that this oscillation appears related to locomotion [11]. However, it is clearly unrealistic that such oscillation would require a constant, explicit external input, and would otherwise collapse to a fixed point (i.e. a static neural pattern). The dynamical structure of this fixed point, from which we wish to drive the system into sustained oscillatory motion, can be investigated through impulse-response experiments.

**Fig 2:**
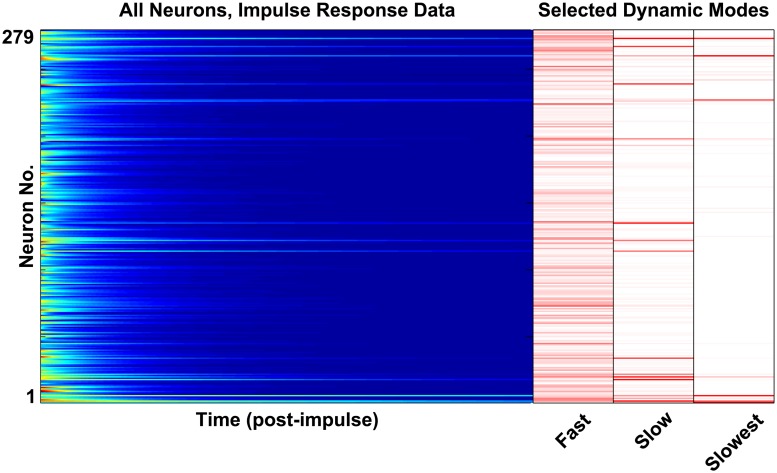
The raster plot at left plots shows a single trial of neuron voltage responses to a random impulse. The nonlinear network dynamics encode six clusters of dynamic modes with timescales spanning three orders of magnitude. In the right panel we plot a subset of the dynamic modes which we extract from these dynamics (the *ϕ* vectors calculated from Eq 14). The modes shown are those with the slowest timescales, later referred to as Modes 4, 5 and 6. One can see the modal dynamics within the raster plot (e.g. one can see traces of the “slow” and “slower” modes, each decaying at a different rate).

In each of 100 separate trials, we model the dynamics of the full somatic nervous system of 279 neurons (where there are 302 neurons total, 282 within the somatic nervous system, and 279 of those which make synaptic connections [30]). We perturbed the system from equilibrium with a short stimulus distributed randomly across all 279 neurons. The system was then allowed to freely decay back to the fixed point, and the decaying neuron voltages were recorded (providing data as shown in Fig 2). We observed that, in all trials, the system decayed back to the same fixed point regardless of input stimulus.

We find that these dynamics are well-described by a few modes (i.e. specific spatial patterns of neural activation), each of which decay exponentially bringing the system back to the fixed point. Applying Dynamic Mode Decomposition to the data gives us both these spatial modes and their decay time constants. Interestingly, we find the following: (1) in all trials the dynamics are well-described by only six modes, (2) DMD gives approximately the same six modes regardless of the random stimulus direction, and (3) the time constants of the modes are well-separated and span three orders of magnitude. Examples of DMD modes and the spatial information which they contain are shown in Fig 2.

These modes can be interpreted as the components of a low-dimensional manifold to which the dynamics are constrained around the fixed point. In other words, an arbitrary stimulus into all 279 neurons can effectively only excite some combination of these six neural patterns. This is what we mean by the fixed point having “low-dimensional structure”.

### Relation of Dynamic Modes to Forward Motion

**Fig 3:**
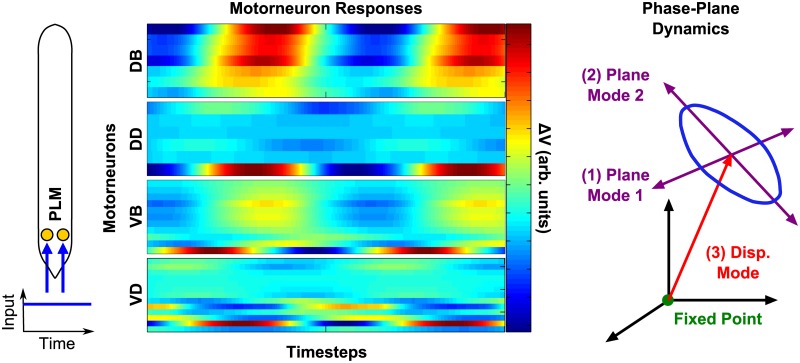
Illustration of the PLM Response as in [11]. Constant stimulation of PLM neurons (corresponding to tail-touch) causes oscillation in body-wall motorneurons. This oscillation can be described as a 2D limit cycle, consistant with the observed 2D body shape dynamics of forward motion [29]. The center of this limit cycle is displaced in the full-dimensional space from the zero-input fixed point. We refer to the two oscillatory modes as the “plane modes”, and the displacement from the fixed point to the center of oscillation as the “displacement mode”.

How does this low-dimensional structure relate to the previously-observed, locomotion-like oscillatory response? To answer this, we note that the PLM response in [11] is characterized by three modes (the “PLM modes”, which define the spatial activity patterns implicated in this response): (1, 2) the two modes defining the plane in which the limit cycle proceeds (the “PLM plane”), which we call the “plane modes”, and (3) the displacement between the equilibrium fixed point and the center of the limit cycle, which we call the “displacement mode”. These modes and their relation to the fixed point and limit cycle are depicted in the “Phase-Plane Dynamics” illustration of Fig 3.

**Fig 4:**
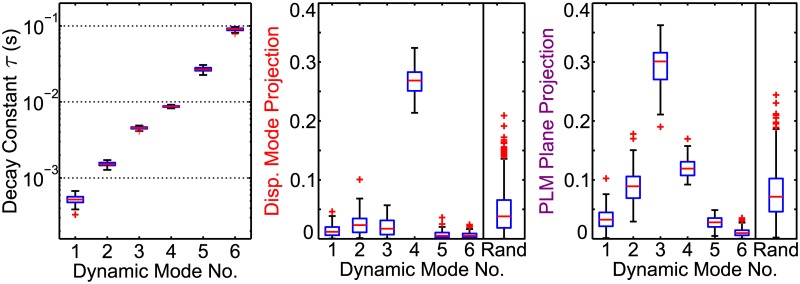
Two of the six dynamic mode clusters correspond to previously-discovered behavioral modes related to locomotion. DMD results from 100 random impulse trials are plotted as box-and-whisker plots showing each mode’s decay constant, as well as projections onto the displacement mode and PLM plane. Projections from 1000 random modes are compared. Timescales of each mode are highly distinct and consistant, and vary over three overs of magnitude. Mode 4 and Mode 3 project strongly onto the Displacement Mode and PLM Plane, respectively. See Tables 1, 2 and 3 for corresponding numerical values.

We investigate the biological meaning of our dynamic modes by calculating their projections onto the PLM modes. This reveals that two of the six dynamic modes correspond to previously-discovered PLM modes. These projection values quantify the similarity between the spatial patterns of the previously-discovered, physiologically meaningful PLM modes and the DMD modes which we inferred from our random impulse trials. Fig 4 shows the magnitude of each dynamic mode’s projection onto the displacement mode and PLM plane. Corresponding numerical values are given in Tables 1, 2 and 3. We compare these against the projections of 1,000 random modes. Both the displacement mode and the PLM Plane have a single dynamic mode projecting strongly onto them. Therefore the low-dimensional structure of the fixed point facilitates responses in both the displacement mode and PLM Plane directions, with a highly distinct timescale for each response.

**Table 1:** 

*τ*	Upper	75th Pct.	Median	25th Pct.	Lower	Spread
Mode 1	0.00067	0.00057	0.00052	0.00048	0.00039	27.6%
Mode 2	0.00172	0.00159	0.00151	0.00146	0.00128	14.6%
Mode 3	0.00489	0.00466	0.00455	0.00445	0.00412	8.5%
Mode 4	0.00920	0.00886	0.00872	0.00856	0.00822	5.6%
Mode 5	0.03065	0.02816	0.02681	0.02568	0.02264	14.9%
Mode 6	0.09705	0.09324	0.09094	0.08736	0.08126	8.7%

Numerical values of the timescales in Fig 4. The consistency of timescales resulting from different trials is given by the “spread”, calculated as (*Upper* − *Lower*)/(2 × *Median*).

**Table 2:** 

Disp.	Upper	75th Pct.	Median	25th Pct.	Lower
Mode 1	0.03858	0.01996	0.01178	0.00597	0.00043
Mode 2	0.06829	0.03442	0.02325	0.01089	0.00120
Mode 3	0.05698	0.03109	0.01701	0.00717	0.00021
Mode 4	0.32392	0.28287	0.26850	0.25110	0.21387
Mode 5	0.02002	0.01063	0.00514	0.00204	0.00013
Mode 6	0.01659	0.00891	0.00507	0.00248	0.00017
Random	0.13578	0.06584	0.03813	0.01850	0.00012

Numerical values of the Displacement Mode Projections in Fig 4.

**Table 3:** 

Plane	Upper	75th Pct.	Median	25th Pct.	Lower
Mode 1	0.07575	0.04464	0.03250	0.02130	0.00117
Mode 2	0.15038	0.10588	0.08901	0.06892	0.02930
Mode 3	0.36243	0.31583	0.30094	0.27032	0.21088
Mode 4	0.15740	0.13069	0.11906	0.10745	0.09184
Mode 5	0.04889	0.03529	0.02791	0.02014	0.00476
Mode 6	0.02739	0.01454	0.00976	0.00588	0.00021
Random	0.18629	0.10235	0.07133	0.04601	0.00214

Numerical values of the PLM Plane Projection in Fig 4.

Mode 4, which projects strongly onto the displacement mode, is particularly interesting. It has the most consistent timescale between trials (see Table 1). Additionally, all other dynamic modes have a particularly *low* projection onto the displacement mode (i.e. a significantly lower median projection than random modes). This suggests that the low-dimensional structure of the fixed point facilitates responses in that direction with a particularly consistent timescale. Thus periodic perturbations of the correct timescale could perturb the system off of the fixed point in the direction of the limit cycle; we discuss these implications further in the Discussion.

### Dynamic Modes Result from Connectivity

The role of the connectome, the experimentally validated network connectivity, was investigated by repeating the perturbation experiments but with randomly changed network connectivities. We considered the following variations: (A) a network with the same degree distribution, with node degrees and connections randomly assigned; (B) random connectivity with the same total number of edges.

**Fig 5:**
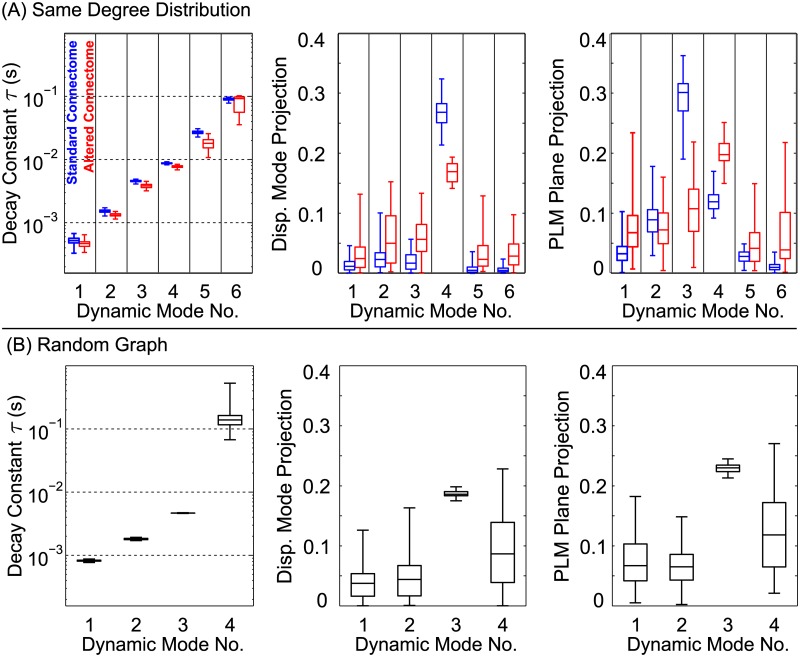
Analysis was repeated for different network connectivities. Each new boxplot takes data from 10 trials of 5 different randomly-generated networks: **(A)** Results from networks generated to have an identical degree distribution. The *τ* distributions for modes 2–6 are statistically different for the altered networks, but still mostly overlap. Projection values **(B)**, however, are both statistically distinct and have very little overlap in some cases (particularly, the Displacement Mode projection of Mode 4, and the PLM Plane Projection of Mode 3). Results from fully random networks. Changing the degree distribution changes the number of modes and their timescales. This shows that the dynamic modes and timescales are encoded by the network’s degree distribution and specific connectivity. This suggests that behavioral dynamics are partially encoded within the connectome itself, the connectivity of which facilitates proprioceptive control.

Results from these cases are summarized in Fig 5. In (A), where the degree distribution is maintained, there are still six modes. However, timescales vary somewhat from the original, and the projections are completely changed. In (B), which has a different degree distribution, the number of modes and all of their properties are qualitatively different. This establishes that the dynamic modes and timescales are encoded by both the network’s degree distribution and specific connectivity.

### Driven Oscillatory Response

We have established that random impulses can drive the system in the direction of the displacement mode. However, given the apparent global stability of the fixed point, an additional mechanism is required for sustained dynamic responses to external input. Proprioception may allow initial perturbations to grow into the desired limit cycle associated, for instance, with forward motion.

We thus investigated the following question: could stretch-receptive proprioceptive feedback within B-class motorneurons give rise to motorneuron oscillations which are qualitatively similar to PLM-driven oscillations? Since locomotion consists approximately of sinusoidal bends propagating along the body [31, 32], it suffices to drive B-class motorneurons with sinusoidal inputs, as determined by their location along the axis of the body and their position on the dorsal/ventral side (noting that the *C. elegans* lays on its side while it crawls [32]).

**Fig 6:**
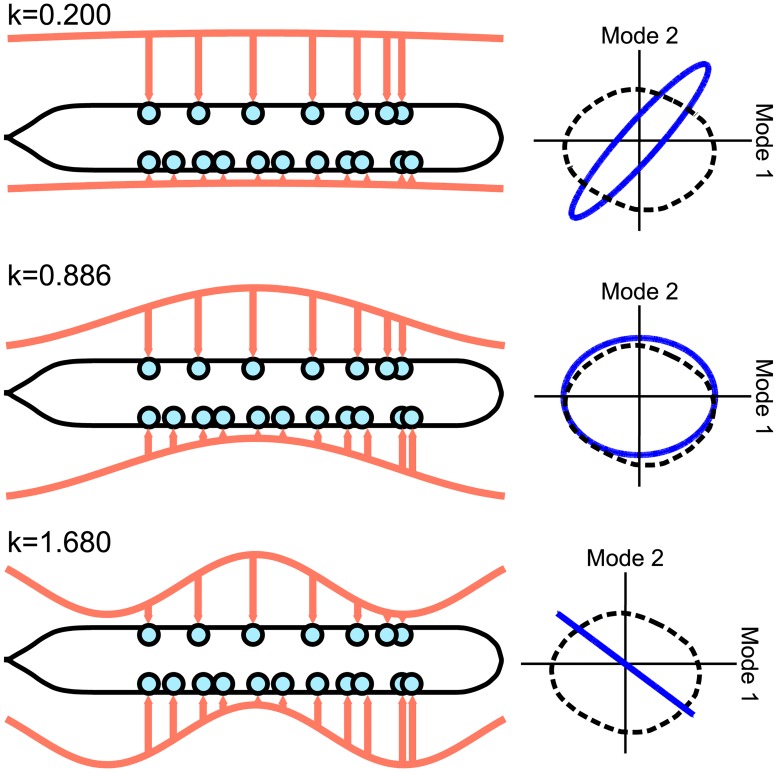
Resulting dynamics from sinusoidally driving B-class motorneurons, approximating proprioception. Dynamics are shown projected onto the PLM plane and plotted as solid blue lines (with the black dashed lines showing the PLM response cycle). Certain spatial wavelengths give rise to qualitatively similar limit cycles.

Fig 6 shows the motorneuron dynamics resulting from different sinusoidal inputs into B-class motorneurons. For certain spatial wavelengths, a limit cycle does occur which is qualitatively similar on the PLM plane. Note that there is a smooth transition between the rows of Fig 6, and that the middle row is the most similar to the PLM-driven cycle (as quantified by the Procrustes distance, a measure of shape similarity used more extensively later in the text). This middle row corresponds to a spatial driving wavenumber of *k* = 0.886 or, equivalently, a spatial driving wavelength (per unit body-length) of λ/*L* = 1/0.886 = 1.13. This value lies well within the range of body-shape wavelengths which, depending on the resistance of the environment, are seen to fall into the range λ/*L* ∈ (0.5, 1.75) (specifically, see Fig 1(e) of [33]). Note that the variation within this range depends directly upon the environmental resistance, which is not included within our model.

Temporal frequency did not have an effect on the shape of the limit cycle, consistent with the experimental observations that the spatial wavelength of *C. elegans* locomotion does not depend on temporal frequency [32]. As discussed further in the Discussion, this suggests that our model, if integrated with a mechanical body model in future work, could be made consistent with a system of feedback-driven oscillations.

### Driven Response on Isolated Subcircuits

**Fig 7:**
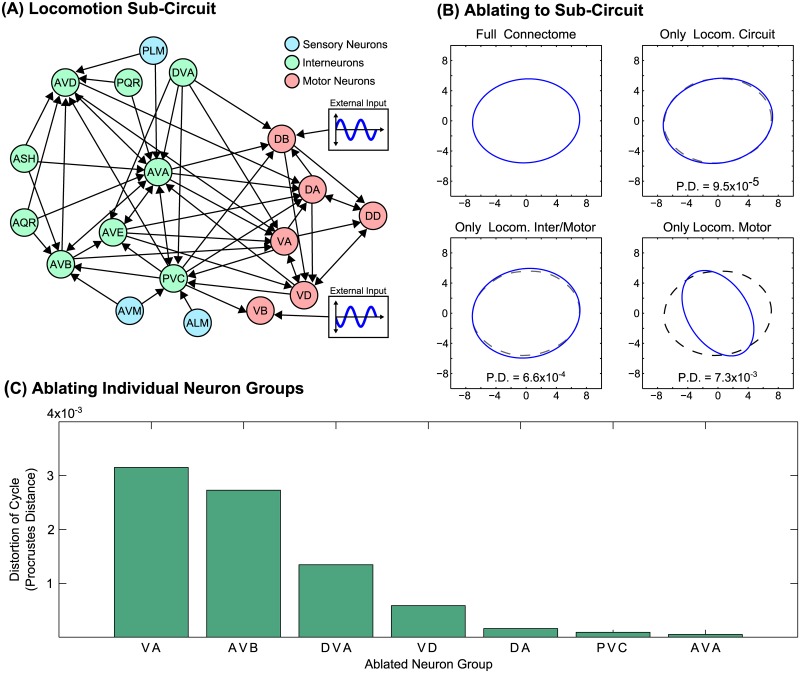
**(A)** The subnetwork of neurons associated with locomotion, as in [34]. Arrows indicate the presence of multiple synaptic connections [30]. **(B)**. The cycle resulting from sinusoidal driving (*k* = 0.886) when the network is reduced to the given subcircuit (i.e. when all other neurons are ablated). The locomotory subcircuit sustains a nearly identical response to the full network, as do the locomotory inter/motorneurons alone, but eliminating the interneurons results in a substantial distortion of the cycle. This distortion from the full-network response is quantified via the Procrustes distance. **(C)** Cycle distortion when specific neurons are ablated. The interneuron ablations leading to the highest level of distortion are known to cause distorted forward locomotion when ablated experimentally (e.g. when ablating AVB [28] or DVA [36]).

An advantage of a full-connectome modeling approach is that it readily enables simulated ablation experiments, in which we may simulate the network with an arbitrary subset of neurons removed. Physiologically, we expect that the response to driving the B-class motorneurons should depend not only upon the neurons which are being driven directly, but also upon associated neurons within the subcircuit of the connectome regulating locomotion, as described in [28, 34–37] and pictured in Fig 7(A). We therefore demonstrate that the experimentally-characterized locomotion subcircuit is, by itself, sufficient to reproduce these results. Furthermore, we elucidate the roles of each of its components.

We therefore investigate the following questions: is the experimentally-characterized locomotion subcircuit by itself sufficient to reproduce these results? If so, what is the relative contribution of its different components?

Fig 7(B) shows the PLM-Plane cycle in response to sinusoidal driving of B-class motorneurons, as in Fig 6 (using a driving wavenumber *k* = 0.886). In addition to calculating this response using the full Connectome, we repeat this simulation with various portions of the network ablated: (1) with all neurons ablated except for the locomotion subcircuit; (2) keeping only the locomotory inter-and motorneurons; (3) keeping only the locomotory motorneurons. We observe that the locomotion subcircuit alone, with the rest of the connectome ablated, reproduces a nearly identical cycle shape. Similarly, when only locomotion inter- and motorneurons are included, the cycle is minimally distorted. However, ablating the locomotory interneurons causes considerable distortion of the cycle, despite the fact that these neurons are not driven directly. Thus we find that these interneurons are crucial in regulating the driven response, which is consistent with evidence for the role of these interneurons in locomotion [28, 36].

We can quantify the degree to which the cycle is distorted by taking the full connectome’s cycle and the ablated connectome’s cycle and computing their Procrustes distance (a measure from statistical shape analysis which increases as the cycle shapes become increasingly dissimilar). The Procrustes Distance (P.D.) of each cycle appears in Fig 7(B), below each distorted cycle. We further used this to calculate the amount of cycle distortion when each individual component of the locomotory circuit was ablated from the full connectome, allowing for the assessment in each neuron’s relative importance in regulating the response. The individual ablations leading to the highest degree of distortion are included in Fig 7(C). Notably, this identifies neurons known to be crucial for the worm’s locomotion ability: experimentally ablating AVB and DVA, for example, are each known to cause significantly distorted forward locomotion [28, 36].

## Discussion

In this manuscript, we have introduced the Dynamic Mode Decomposition as a diagnostic tool to characterize impulse-response experiments on a nonlinear networked system. This revealed that the network is structured to generate a low-dimensional response at distinct timescales ranging over several orders of magnitude, and that two of these dynamic modes are related to the previously-characterized “forward motion” response to PLM-stimulation. It is possible that proprioceptive feedback could *sustain* a limit cycle but not be sufficient to bring the system to said limit cycle from the equilibrium fixed point. In other words, the limit cycle would need to be “jumpstarted”, with a separate mechanism transporting the system from the fixed point near to the cycle. Were this the case, it would suggest a physiological purpose for the low-dimensional fixed-point structure which we detect: stimuli of the correct timescales could selectively perturb the system towards the limit cycle, to a point from which the proprioceptive feedback could be effective. In this view, it is interesting and suggestive that Mode 4, associated with the Displacement Mode, has the most tightly-constrained timescales of all the modes.

Repeating this analysis for different connectivities suggested that these dynamic modes and timescales are encoded by both the network’s degree distribution and specific connectivity. A random graph, with the same number of nodes and connections but a different degree distribution, leads to a completely different number of modes. This suggests that the number of dynamical timescales is encoded by the degree distribution, as six timescales are recovered for any network with the same degree distribution. However, the specific timescale values and the neuronal makeup of these modes is not preserved. The degree to which each mode projects onto our biophysiologically-relevant directions, and with what specific dynamical timescale, depends on the specific wiring of the connectome. Thus behavioral dynamics are partially encoded within the connectome itself, the connectivity of which facilitates proprioceptive control. Said another way, the stereotyped worm connectome seems to be optimized for its behavioral repertoire.

The usefulness of these insights as they apply to the actual system, however, depend on the model’s compatibility with a framework of proprioception-generated oscillation. Thus we further show that sinusoidal input into the putatively proprioceptive B-class motorneurons does, indeed, drive a limit cycle at certain spatial wavelengths, consistent with the spatial wavelengths seen experimentally. Given that the worm crawls with a sinusoidal body shape [31, 32], this suggests that motorneuron proprioception could indeed drive the limit cycle, which in turn could drive sinusoidal movement. A proprioceptive mechanism such as this is necessary for sustained dynamic responses to external input. Furthermore, we showed that the motor subcircuit alone is capable of sustaining these results, and that this circuit’s interneurons are crucial to regulating the response despite not being driven directly. Indeed, we found that the interneurons which were most important to regulating the response within our simulation were those which have been shown to have this exact role experimentally. Despite this apparent consistency, the development of such a feedback rule remains nontrivial. Without a coupled biomechanical model that includes muscle activation, any feedback rule which we might implement on the present model would be no less artificial than our direct sinusoidal stimulus, which is biophysiologically reasonable.

However, modeling the worm’s body and environment is ultimately crucial to fully understanding its behavior [7, 10, 33, 38–40]. This study prescribes multiple studies for future computational connectome models which *are* fully integrated with biomechanical body and environmental models (as exemplified by projects such as OpenWorm [41]). Specifically, it introduces the following questions: (1) When motorneuron proprioception and other external feedback is turned off within a model, does the system decay into a fixed point? If so, an identical study can be performed to probe that fixed point’s low-dimensional structure. (2) Do the dynamic modes relate to the oscillatory dynamics which occur during locomotion? (3) If proprioception/feedback is turned back on while the system is in its fixed point, does the system proceed into a spontaneous limit cycle, and if so, how? Is periodic noise or other stimulation of a specific timescale necessary for such a transition?

More broadly, this work demonstrates the utility of Dynamic Mode Decomposition in relating the specific connectivity of a network to the multi-scale, low-dimensional structure of its dynamical responses. The methods of this manuscript are able to directly relate connectivity to dynamics even for large, nonlinear networked systems. Future work will further investigate this relationship, with implications for the design of nonlinear networks.

## Methods

### Modeling the *C. elegans* Connectome

Our model for the *C. elegans* simulates the neuronal dynamics of its full connectome, as obtained from [30]. This network consists of the 279 somatic neurons which make synaptic connections. Between these neurons, there are 6393 synaptic connections and 890 gap junctions, and the connectivity between neurons cannot be considered sparse. Further details on the network’s structural properties are available in [30], and further information, including about putative functions of individual neurons, is collected within WormAtlas [42].

Experiments show that many neurons in the organism are effectively isopotential, such that membrane voltage is a meaningful state variable [43]. Wicks et al. constructed a single-compartment membrane model for neuron dynamics [44], which we later adapted to incorporate recent connectomic data [11]. We assume that the membrane voltage dynamics of neuron *i* is governed by:

$$\begin{array}{c} C_{i}\dot{v_{i}}=-G_{i}^{c}\left(v_{i}-E_{cell}\right)-I_{i}^{Gap}\left(v\right)-I_{i}^{Syn}\left(v\right)+I_{i}^{Ext}. \end{array}$$ (1)

The parameter *C*_*i*_ represents the whole-cell membrane capacitance, $G_{i}^{c}$ the membrane leakage conductance and *E*_*cell*_ the leakage potential of neuron *i*. The external input current is given by $I_{i}^{Ext}$. Neural interaction via gap junctions and synapses are modeled by the input currents $I_{i}^{Gap}\left(v\right)$ (gap) and $I_{i}^{Syn}\left(v\right)$ (synaptic). Their equations are given by:

$$\begin{array}{c} I_{i}^{Gap}=\sum_{j}G_{ij}^{g}\left(v_{i}-v_{j}\right) \end{array}$$ (2)

$$\begin{array}{c} I_{i}^{Syn}=\sum_{j}G_{ij}^{s}s_{j}\left(v_{i}-E_{j}\right) \end{array}$$ (3)

We treat gap junctions between neurons *i* and *j* as ohmic resistances with total conductivity $G_{ij}^{g}$. We assume that $I_{i}^{Syn}$ is also modulated by a synaptic activity variable *s*_*i*_, which is governed by

$$\begin{array}{c} \dot{s_{i}}=a_{r}\phi \left(v_{i};\kappa ,v_{th}\right)\left(1-s_{i}\right)-a_{d}s_{i}. \end{array}$$ (4)

Here *a*_*r*_ and *a*_*d*_ correspond to growth and decay time, and *ϕ* is the sigmoid function *ϕ*(*v*_*i*_;*κ*, *v*_*th*_) = 1/(1 + exp(−*β*(*v*_*i*_ − *v*_*th*_))).

Simulations were performed in MATLAB via Euler’s method, using timesteps of *h* = 10^−6^s. The data was downsampled by recording *v*(*t*) every Δ*t* = 3 × 10^−5^s, yielding a data matrix:

$$\begin{array}{c} V=\left[\begin{array}{cccc} | & | & & |\\ v(t_{1}) & v(t_{2}) & \cdots & v(t_{m-1})\\ | & | & & | \end{array}\right], \end{array}$$ (5)

where *t*_*k*+1_ − *t*_*k*_ = Δ*t*. The value of Δ*t* was chosen to be sufficiently low so as to not affect the outcome of the analysis.

### Model Parameters

We keep all parameter values from [11]. The number of gap junctions $N_{ij}^{g}$ and number of synapses *Nij*^*s*^ are taken from the large component of the full connectome, i.e. the 279 neurons as considered in Varshney, et al. [30]. Each individual synapse and gap junction is assigned an equal conductivity of *g* = 100pS (such that $G_{ij}^{g}=g\cdot N_{ij}^{g}$ and $G_{ij}^{s}=g\cdot N_{ij}^{s}$). The values of cell membrane conductance and capacitance are *G*^*c*^ = 10pS and *C* = 1pF. The synaptic growth and decay constants are kept as *a*_*r*_ = 1 s^−1^ and *a*_*d*_ = 5 s^−1^. All neurons are modeled as identical except for their connectivity and the assignment of them as excitatory or inhibitory (where *E*_*j*_ will have one of two values corresponding to these classes).

### Random Perturbations

For each random perturbation simulation, a random external input *I*^*Ext*^ was applied to all neurons for a duration of 10^−5^s, after which the system was allowed to decay. Output was recorded from all neurons after the cessation of input. Each $I_{i}^{Ext}$ was drawn from a Gaussian distribution, after which the total *I*^*Ext*^ was then normalized to have a fixed total input amplitude of |*I*^*Ext*^| = 10mA.

### Dynamic Mode Decomposition

This section describes the method of Dynamic Mode Decomposition [45–52], which we apply to our simulated neural voltage data **V**. Specifically, we use it to relate the voltages at timestep *t*_*k*_ to the following timestep *t*_*k*+1_ as follows:

$$\begin{array}{c} v\left(t_{k+1}\right)\approx Av\left(t_{k}\right), \end{array}$$ (6)

where $A\in R^{n\times n}$ is the linear operator which is the best-fit solution for all pairs. Note that this does not imply that the underlying dynamics are linear; DMD is connected to nonlinear dynamical systems through the Koopman operator [50]. We can express this relationship in matrix form by constructing two data matrices $X\in R^{n\times (m-1)}$ and $X^{\prime }\in R^{n\times (m-1)}$ as follows:

$$\begin{array}{c} X=\left[\begin{array}{cccc} | & | & & |\\ v(t_{1}) & v(t_{2}) & \cdots & v(t_{m-1})\\ | & | & & | \end{array}\right], \end{array}$$ (7)

$$\begin{array}{c} X^{\prime }=\left[\begin{array}{cccc} | & | & & |\\ v(t_{2}) & v(t_{3}) & \cdots & v(t_{m})\\ | & | & & | \end{array}\right]. \end{array}$$ (8)

This allows us to write Eq (6) as:

$$\begin{array}{c} X^{\prime }\approx AX. \end{array}$$ (9)

The dynamic mode decomposition of the data matrices (**X**, **X**′) is given by the leading eigendecomposition of the matrix **A**, which is defined as follows:

$$\begin{array}{c} A=X^{\prime }X^{†}, \end{array}$$ (10)

where † denotes the Moore-Penrose pseudoinverse [47]. The pseudoinverse of **X** can be found by calculating its singular value decomposition, truncated at *r* singular values:

$$\begin{array}{c} X\approx \tilde{U}\tilde{\Sigma }{\tilde{V}}^{*}. \end{array}$$ (11)

Here * denotes the complex conjugate transpose, $\tilde{U}\in R^{n\times r}$ and $\tilde{V}\in R^{m-1\times r}$ are matrices with orthonormal columns, and $\tilde{\Sigma }\in R^{r\times r}$ is diagonal. The diagonal entries of **Σ** are the singular values, and are proportional to the percentage of energy within each mode. We choose the smallest set of *r* modes which capture 99% of the energy.

We can thus approximate the linear operator **A** as follows:

$$\begin{array}{c} A\approx \bar{A}=X^{\prime }\tilde{V}{\tilde{\Sigma }}^{-1}{\tilde{U}}^{*}. \end{array}$$ (12)

We are interested in the dynamics projected upon the lower-dimensional subspace as defined by the first *r* columns of $\tilde{U}$. Rather than calculating the *n* × *n* matrix $\bar{A}$, we project onto the low-dimensional subspace to calculate the *r* × *r* reduced order operator $\tilde{A}$:

$$\begin{array}{c} \tilde{A}={\tilde{U}}^{*}X^{\prime }\tilde{V}{\tilde{\Sigma }}^{-1}. \end{array}$$ (13)

The eigendecomposition $\tilde{A}W=W\Lambda$ gives the eigenvectors **w**_j_ and eigenvalues λ_*j*_ of the reduced-order system. The eigenvalues are equal to those of the full-dimensional $\bar{A}$, and the corresponding eigenvectors can be used to exactly calculate the full-dimensional dynamic modes of the system [47]. For λ_*j*_ ≠ 0, the dynamic mode corresponding to **w**_j_ is:

$$\begin{array}{c} \phi_{j}=X^{\prime }\tilde{V}{\tilde{\Sigma }}^{-1}w_{j}. \end{array}$$ (14)

The DMD modes take the eigenvectors of the reduced-order system and project them back to the full-dimensional space. In our system, this means that a dynamic mode *ϕ*_*j*_ will be a vector of length 279, with each element corresponding to the relative activation of a neuron within each mode. Since these dynamic modes correspond to the eigenvectors of the low-dimensional system, the modes give the dynamically-decoupled low-dimensional patterns which will exponentially grow/decay and/or oscillate with timescales given by their respective eigenvalues λ_*j*_. The state of the system just after perturbation may be written in terms of these modes:

$$\begin{array}{c} v\left(t=0\right)\approx \sum_{j=1}^{r}c_{j}\phi_{j}. \end{array}$$ (15)

After *k* timesteps Δ*t* = *t*_*k*+1_ − *t*_*k*_, the system will then be within the state:

$$\begin{array}{c} v\left(t_{k}\right)\approx \sum_{j=1}^{r}c_{j}\lambda_{j}^{k}\phi_{j}. \end{array}$$ (16)

We can also write the solution for an arbitrary time *t* as:

$$\begin{array}{c} v\left(t_{k}\right)\approx \sum_{j=1}^{r}c_{j}\phi_{j}\exp\left(-t/\tau_{j}\right). \end{array}$$ (17)

The continuous decay constant *τ*_*j*_ can be directly calculated from the DMD eigenvalue as follows:

$$\begin{array}{c} \lambda_{j}=\exp\left(-\Delta t/\tau_{j}\right)\;\to \;\tau_{j}=\frac{-\Delta t}{\ln\left(\lambda_{j}\right)}. \end{array}$$ (18)

In general, *τ*_*j*_ may be complex with any sign. Clearly, Re(*τ*_*j*_) > 0 will lead to exponential decay, Re(*τ*_*j*_) < 0 will lead to exponential growth, and Im(*τ*_*j*_) ≠ 0 will lead to oscillation. For all trials within this manuscript, however, the resulting *τ*_*j*_ values were seen to be positive and real, due to the dynamics of the dataset being well described by non-oscillatory decay.

### Dynamic Mode Properties

The properties of the resultant modes are summarized in the boxplots of Figs 4 and 5. These were generated from MATLAB function boxplot.m. Default settings are used in Fig 4, and in Fig 5 the settings are changed such that no points are treated are outliers.

The PLM modes are calculated by taking the singular value decomposition of the PLM-driven limit cycle, as in [11]). The PLM modes include the displacement mode **d** and plane modes **p_1_**, **p_2_**, as illustrated in Fig 3. Each of these modes are a vector of length 279, with each element corresponding to the relative activation of a neuron’s membrane voltage. Each represents a neural pattern which is dynamically active which the system while it is in the PLM-driven limit cycle, which is argued in [11] to represent a neural proxy for forward locomotion.

The projection metrics are defined as the projections of each dynamic mode vector *ϕ***_i_** onto the displacement mode vector **d** and onto the PLM Plane {**p_1_**, **p_2_**}. Specifically:

$$\begin{array}{c} \text{Disp}.\;\text{Mode}\;\text{Projection}=\phi_{i}\cdot d \end{array}$$ (19)

$$\begin{array}{c} \text{Plane}\;\text{Projection}=\sqrt{{\left(\phi_{i}\cdot p_{1}\right)}^{2}+{\left(\phi_{i}\cdot p_{2}\right)}^{2}} \end{array}$$ (20)

The random projections in Fig 4 are calculated similarly, but using a randomly-generated mode in place of an actual DMD mode. For each random mode, each element is chosen from a Gaussian distribution and the mode is then normalized.

### Altered Connectomes

We repeated our Dynamic Mode Decomposition analysis for altered networks with (A) the same degree distribution, but altered specific connectivity, and (B) random graphs with the same total number of connections. For both cases, we generated 5 distinct altered networks, for which we performed 10 impulse-response trials each. We calculated the DMD results (decay constants *τ* and displacement mode/PLM plane projections) for each set of trials. Thus for both (A) and (B) we obtained 5 sets of 10 *τ*/projection values each, each set corresponding to a different altered network. Fig 3 plots the distributions of results for *all* altered networks of a given type (i.e. plotting all 50 values for each mode).

All altered connectomes with the same degree distribution, in all of their random-impulse response trials, yield six dynamic modes, as shown in Fig 3(A). This is the same number modes as is produced by the standard connectome, and thus the distributions of *τ* and projection values may be directly compared. We wish to determine if the results which we obtain from the altered connectome are statistically different from those which we obtain from the standard connectome. For each of the 5 altered connectomes, we compare the altered and standard *τ*/projection distributions using the two-sample Kolmogorov-Smirnov test, with the null hypothesis that they are from the same distribution (computed in MATLAB using the built-in function kstest2.m). The maximum p-values for each distribution from the set of tests is shown in Table 4. At a significance level of *p* = 0.05, we can conclude that altering the specific connectivity alters the following results: the *τ* values of Modes 2, 3, 4, 5 and 6; the displacement mode projections of Modes 4, 5 and 6; and the plane mode projections of Modes 3, 4 and 6.

**Table 4:** 

Maximum p-Values	*τ* Dists.	Disp. Dists.	Plane Dists.
Mode 1	0.2885	0.9987	0.8608
Mode 2	0.0004	0.2233	0.9590
Mode 3	1.796 × 10^−5^	0.289	8.126 × 10^−9^
Mode 4	1.776 × 10^−8^	3.663 × 10^−9^	8.126 × 10^−9^
Mode 5	5.120 × 10^−6^	0.0070	0.0938
Mode 6	0.0107	0.0131	0.0107

Maximum *p*-values, for the null hypothesis that the *τ*/projection values for the Altered Connectomes in Fig 3(A) are from the same distribution as those for the Standard Connectome. Specifically, we compare the two distributions using the two-sample Kolmogorov-Smirnov test. At the *p* = 0.05 level we can conclude that the following distributions are significantly different after changing the specific connectivity: the *τ* values of Modes 2, 3, 4, 5 and 6; the displacement mode projections of Modes 4, 5 and 6; and the plane mode projections of Modes 3, 4 and 6.

All of the random graphs, in all of their trials, yielded four dynamic modes, as shown in Fig 3(B). As is apparent in the Fig, Mode 3 is notable for having very consistent projection values onto each mode. Note, however, that this mode is trivial: it is simply equal for all nodes (i.e. it is the vector $\phi_{i}=1/\sqrt{279}$ for each of the *i* ∈ (1, 279) neurons). This mode, though trivial, will have a higher projection value than will sparser modes which select the “wrong” neurons.

### Sinusoidal Driving of B-Class Motorneurons

In approximating proprioceptive input, we sinusoidally drove all B-class motorneurons, using an external input of the following form:

$$\begin{array}{c} I_{i}^{Ext}=\pm A\sin\left(\omega t-kx\right) \end{array}$$ (21)

Input sign was given based on the dorsal/ventral location of the motorneuron. Input amplitude *A* affected only the amplitude of the cycle and was set at *A* = 30 Arb. Units to yield a qualitatively similar cycle amplitude. Temporal frequency *ω* appeared to affect the response only by changing the cycle period. Spatial wavelength *k* varied between trials (as shown in Fig 6). *x* was assigned to each neuron based on its soma position.

Soma position data originates from [30], and was retrieved from the “Neuronal Wiring” section of WormAtlas [42]. The use of the soma position is a simplification: proprioception in B-class motorneurons is believed to be due to stretch reception within the long axons posterior to the soma [9].

The plane dynamics plotted in Figs 6 and 7 were calculated by taking the projection of the full-dimensional dynamics **v**(*t*) onto the plane modes **p_1_** and **p_2_**. This gives the cycle dynamics projected into the low-dimensional space, as in [11].

### Regulation of Driven Cycle by Subcircuits

We also calculated the response to sinusoidal driving of B-class motorneurons for networks with various neurons removed. We were particularly interested in the role of the motor subcircuit in regulating the response. As in [34], we take the following neuron groups as comprising the motor circuit:

- *Motor Circuit Sensory Neurons*: ALM, AVM, PLM
- *Motor Circuit Interneurons*: AVA, AVB, AVD, AVE, ASH, AQR, DVA, PVC, PQR
- *Motor Circuit Motorneurons*: DA, DB, DD, VA, VB, VD

“Simulated ablation” of a neuron is done similarly to how it was performed in [11], i.e. by simply removing the connections of selected neurons. In other words, we use the same model, with the connectivity data altered such that:

$$\begin{array}{c} G_{ij}^{g}=G_{ij}^{c}=0\;\text{if}\;i\;\text{or}\;j\;\text{ablated} \end{array}$$ (22)

Specifically, we calculate the driven limit cycle response, projected onto the PLM plane, for the following ablation sets:

1. Ablating all neurons *except* those in the motor circuit
2. Ablating all neurons *except* interneurons and motorneurons in the motor circuit
3. Ablating all neurons *except* motorneurons in the motor circuit
4. Ablating all neuron groups in the motor circuit individually (except for B-class motorneurons)

### Procrustes Measure of Cycle Similarity

*Procrustes Distance* (PD) measures the dissimilarity between shapes, and we use it to quantify the similarity between the shapes of the limit cycles pre- and post-ablation. We use the function procrustes.m from MATLAB’s Statistics and Machine Learning Toolbox. We collect *N* data points from each trajectory and annotate their (*x*, *y*) coordinates in a (2 × *N*) shape matrix *S*. The PD between two distinct shapes *S*_*A*_ and *S*_*B*_ is then given by

$$\begin{array}{c} PD=\min_{b,R,c}{||S_{B}-b\cdot S_{A}\cdot R+\vec{c}||}_{2}. \end{array}$$ (23)

In other words, it finds the optimal (2D) rotation matrix *R*, scaling factor *b* > 0, and translation vector $\vec{c}$ to minimize the sum of the squares of the distances between all points. Intuitively, it compares the shapes of the cycles while ignoring any translation, rotation, or scaling. Note that trajectories must be pre-processed to extract data points for a single period of the cycle. Cycles are also interpolated using MATLAB’s spline.m function to ensure that they have the same number of data points. Both limit cycles must also be phase-aligned, which we achieve by finding the relative phase that minimizes the Procrustes Distance. This results in a score which increases as the post-ablation cycle becomes increasingly dissimilar in shape to the pre-ablation cycle.
